# Travel-time accessibility and adaptive spatial planning solutions for the healthcare system

**DOI:** 10.1038/s44401-025-00028-1

**Published:** 2025-07-03

**Authors:** Jose Balsa-Barreiro, Sérgio F. A. Batista, Gaby J. Hannoun, Monica Menendez

**Affiliations:** 1 Division of Engineering, New York University AD, Abu Dhabi, UAE; 2 Center for Interacting Urban Networks (CITIES), New York University AD, Abu Dhabi, UAE; 3 Department of Civil and Environmental Engineering, Imperial College London, London, UK; 4 Department of Geography, University of Santiago de Compostela, Santiago, Spain

**Keywords:** Socioeconomic scenarios, Health care, Geography, Social sciences, Geography, Social policy

## Abstract

Ensuring equitable healthcare access remains a significant challenge, particularly in rural areas where aging populations face increasing barriers due to facility closures. This study employs a GIS-based spatial network model to assess hospital accessibility, integrate road characteristics, and estimate travel times across all officially registered population nodes in a region. Analyzing disparities by age composition, we identify high-risk exclusion areas and propose an optimized model prioritizing vulnerable populations. Through four optimization scenarios, we evaluate the impact of hospital reductions on territorial accessibility. Findings reveal significant gaps, disproportionately affecting elderly and remote communities. Strategic hospital redistribution can enhance system efficiency and equity. This research highlights the value of GIS-based approaches in healthcare planning, providing data-driven strategies for optimizing hospital distribution and informing evidence-based policymaking.

## Introduction

Global demographic shifts redefine healthcare access, with urbanization and aging as key drivers. The global urban population has increased from 15% in 1900 to 55% today, projected to reach 70% by 2050^1^. Meanwhile, the proportion of people 65 years and older has grown from 5% in 1960 to 9.6% in 2021 (761 million people) and is expected to double to 16% by 2050^2^. These trends are even more pronounced in Western countries, where urbanization rates exceed 80% and could reach 90% by 2050, while it is projected that older populations will reach 30% in Europe and 22% in the United States^3^. Japan exemplifies this demographic shift, with 91% urbanization and 29% of its population over 65 years old, projected to increase to 38% by 2050 ^4^.

Disparities in healthcare access are due to social and spatial inequalities, particularly in rural areas where accessibility is limited^5–8^. Studies have consistently shown that travel time is a crucial determinant of access to healthcare, directly influencing health outcomes^9,10^. In remote areas, long distances to healthcare facilities lead to higher mortality rates, particularly among the elderly and chronically vulnerable populations^11–13^. The issue is especially pronounced in sparsely populated regions^14–17^, where large distances exacerbate inequalities. In this context, strategies such as hospital redistribution^15^, emergency fleet optimization^18^, emerging technologies such as connected vehicles^19^ and telemedicine^20^ have been proposed to mitigate accessibility issues.

From a spatial perspective, urban areas benefit from dynamic economies of scale, which shorten the distance between people and services. In contrast, rural regions face depopulation and aging, making it harder to maintain essential services, including healthcare^21,22^. The *World Health Organization*^23^ identified transportation costs as a major barrier to access to healthcare for low-income elderly populations, exacerbating social isolation and reduced mobility^24,25^. Addressing these disparities aligns with *Sustainable Development Goals* (SDGs) 3, 10, and 11 of the *2030 UN Agenda*, which emphasizes equitable access to healthcare.

Geographic Information Systems (GIS) play a critical role in quantifying spatial disparities on healthcare accessibility, optimizing the spatial distribution of medical services, and supporting data-driven planning for more equitable healthcare systems. Scholars have developed several methods to analyze spatial accessibility to healthcare services, such as floating catchment areas (including their respective versions)^26^, kernel density estimation^27^, gravity-based models^28^, multi-criteria decision analysis^29^, spatial interaction methods such as the Huff model^30^, network analysis^31^, and agent-based models^32^, among others^33–35^. These methods have been applied using different measures, including actual distances (Euclidean or network-based) and travel times^36,37^. The latter is the most commonly used metric, as it integrates factors such as physical distance, topography, infrastructure quality, and existing mobility services within the studied region^6,7,10,15,38,39^.

In this paper, we analyze travel time accessibility to healthcare facilities (i.e. hospitals) across a region. For that, we implement a spatial network linking population nodes and hospitals via road infrastructure. Our analysis first evaluates the current scenario, assigning each population node to the nearest hospital. The following is taken to establish a baseline assessment of spatial accessibility: (i) it is assumed that all individuals have access to a private vehicle or public transport, consistent with previous large-scale studies^10^; (ii) people always visit the nearest hospital, although real-world choices depend on hospital specialization, perceived quality, and personal preferences; (iii) traffic congestion effects are not considered in travel times, although it will be incorporated into future work; and (iv) hospitals are assumed to have uniform capacity and service offerings, although future work will integrate hospital-specific constraints and services offered.

Beyond the current scenario, we simulate four progressive hospital redistribution strategies aimed at minimizing travel times and identifying vulnerable areas, particularly in aging rural regions. These scenarios include a gradual reduction in hospital numbers to account for the aging population and resource constraints. Conducted within a GIS environment, this study integrates spatial network analysis and facility location modeling to illustrate systematic spatial inequalities in hospital access, particularly among elderly populations. Even under idealized conditions, where everyone has access to a vehicle and there is no congestion, the elderly remain disproportionately disadvantaged. Our findings highlight the need to reconsider how spatial optimization models define accessibility criteria, offering data-driven insights for policymakers to optimize healthcare access under both current and adaptive spatial planning frameworks.

## Results

This section presents the results structured into four main parts. First, we introduce the geographical area and datasets. Second, we evaluate travel-time accessibility under the current hospital distribution and under alternative configurations that optimize spatial distributions in the context of potential reductions in the number of hospitals. Third, we conduct a comparative analysis of the different configurations, using coverage-related indicators to evaluate their impact on accessibility. Finally, we examine how changes in hospital distribution affect accessibility patterns and demographic disparities, highlighting implications for equitable access to healthcare services.

### Geographical, demographic, and transport landscape of Galicia

#### Study area and settlement patterns

**Fig. 1:**
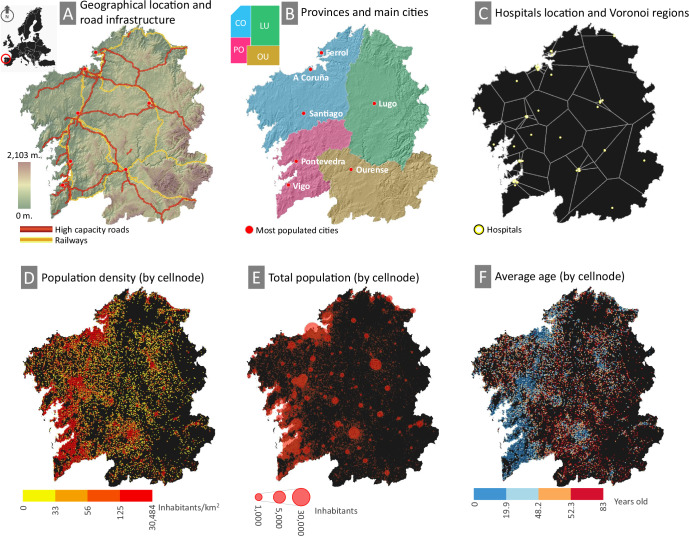
Characterization of the study area and datasets. The upper panels present general data: **A** Geographic location, major transportation routes, and topography, displaying only high-capacity roads and railways, each with an approximate actual length of 1100 km. **B** Administrative division by province and location of the most populated cities. **C** Spatial distribution of hospitals and theoretical coverage based on the Voronoi diagram. The lower panels display data extracted from the population grid at the cell level: **D** population density by quantiles; **E** total population by node size; and **F** average age by quantiles.

Galicia, a region in northwestern Spain, covers 30,000 km^2^ and is home to 2.7 million inhabitants (Fig. 1a). This represents 5.5% of Spain’s population and 5.8% of its land area, with a population density of 91.3 individuals/km^2^—slightly below the national average of 95.3^40^. Despite its modest population share, Galicia has a unique settlement structure, containing almost half of the total population units of Spain^41^.

Historically, the model of rural and agrarian settlement in Galicia featured dispersed populations clustered around small town centers serving as commercial hubs^42^. However, since the late 20th century, rapid urbanization has shifted populations toward the *Atlantic Corridor* along the western coastline. This corridor now includes five of Galicia’s seven major cities: Vigo and A Coruña (~300,000 residents each), Santiago, Ferrol, and Pontevedra (Fig. 1B). In the interior, only Ourense and Lugo (~100,000 residents each) remain significant urban centers, while suburban areas and former city centers have developed into medium-sized cities^43^.

The *Atlantic Corridor* dominates Galicia’s urban hierarchy, shaped by natural growth and migration of the working age population. Of the four administrative provinces of the region, it crosses the two most populous: A Coruña (1.1 million residents) and Pontevedra (936,000). On the other hand, Lugo (290,000) and Ourense (291,000) experience the opposite trend, with depopulation and an increasingly elderly demographic. Eastern Galicia is particularly affected, with smaller and dispersed population nodes and an increasing average age further from urban centers. In general, seniors (65 years and older) represent 26.1% of the total population of Galicia, with the largest proportions in Lugo (29.9%) and Ourense (31.9%), compared to A Coruña (25.5%) and Pontevedra (23.6%)^40^.

These spatial patterns have direct implications for accessibility to the healthcare system. Although urban areas benefit from concentrated healthcare services, rural and aging populations face longer travel times and limited mobility, exacerbating spatial inequalities in hospital access.

### Demographic distribution and population data set

The *Galician Statistical Office* provides high-resolution population census data using a 1 km^2^ grid, consisting of 30,773 cells^44^. Among these, 10,545 cells are populated, with an average of 248 residents per cell. Since the grid does not perfectly align with administrative boundaries, 2228 cells were cropped to improve geoprocessing precision and computational efficiency.

Each grid cell contains total population data and age distribution in three groups: young (under 16 years), adults (16–64), and elderly (over 64). Of the 10,545 populated cells, 6356 report young population data, 934 of which have a value of 0; 9550 contain adult population data; and 7181 include elderly population data, with 2 having a value of 0. The remaining cells either have incomplete information or data are not available for cells with fewer than 20 residents or age groups to comply with the *European General Data Protection Regulation* (GDPR)^45^.

In general, the grid captures 90.9% of the total population of Galicia, although the representation varies by age group: 95.5% for young people, 92% for adults, and 86.2% for elderly^46^. The representation is higher in the western provinces, A Coruña and Pontevedra, where it exceeds 90%, while in the eastern provinces, Lugo and Ourense, it is lower at 75.1% and 83.2%, respectively. The representation of older people is particularly uneven, ranging from 94.2% in Pontevedra to 66.4% in Lugo (Table 1). A visualization of selected parameters from this data set is shown in Fig. 1D–F.

**Table 1:** Percentage of population captured within the grid for each province and age group relative to the actual population

Percentage (2022)	Galicia	A Coruña	Lugo	Ourense	Pontevedra
Young pop.	95.5	96.3	86.2	92.0	98.2
Adult pop.	92.0	93.8	77.6	85.4	96.5
Elderly pop.	86.2	89.8	66.4	76.5	94.2
Total pop.	90.9	93.0	75.1	83.2	96.1

### Healthcare infrastructure and hospital facilities

Healthcare services in Galicia are structured into seven distinct zones according to potential demand, proximity to medical facilities and demographic and political-administrative criteria. The eastern provinces, Lugo and Ourense, each form a single healthcare zone, while the more densely populated western provinces are divided into five smaller zones. Hospitals form the backbone of the regional healthcare system, offering emergency services and specialized care 24/7. The region has 79 hospitals, mainly concentrated in urban areas, particularly along the *Atlantic Corridor*, where the population density is the largest^47^. The service areas of these hospitals can be delineated through various methodologies such as the *Voronoi diagram*, the *Gravity model*, the *Huff model*, and more sophisticated approaches incorporating service flows between areas^48^ to define functional regions more precisely.

For a theoretical estimation of hospital service areas, the *Voronoi diagram* provides a valid methodology^49^. This method divides the region into distinct areas, each centered around a hospital, ensuring that any point within a given area is closer to its respective hospital than to any other. Given two nodes, *a* = (*a*_*x*_, *a*_*y*_) and *b* = (*b*_*x*_, *b*_*y*_), the Euclidean distance is $d(a,b)=\sqrt{{({a}_{x}-{b}_{x})}^{2}+{({a}_{y}-{b}_{y})}^{2}}$. This extends to a set of input data corresponding to a point grid, where *A* = {*a*_1_, *a*_2_, …, *a*_*n*_}, with *n* being the total number of points in the plane. Although simple, this method provides a clear and straightforward approach to defining service areas based solely on proximity criteria.

The set of all points closer to *a*_*i*_ ∈ *A* than to any other *a*_*j*_ (*j* ≠ *i*) defines the Voronoi cell *V*_*a*_(*a*_*i*_) of *a*_*i*_, expressed as

1 $${V}_{a}({a}_{i})=\{b\in {{\mathbb{R}}}^{2}| d(b,{a}_{i}) < d(b,{a}_{j}),\forall j\ne i\}$$

The complete *Voronoi diagram* Vor(*A*) of *A* is then defined as the collection of all Voronoi cells:

2 $$\,{\rm{Vor}}\,(A)=\{{V}_{a}({a}_{i})| i=1,2,\ldots ,n\}$$

In this case study, the region is divided into 79 Voronoi polygons, each representing the theoretical service area of an individual hospital. A clear spatial trend emerges where the polygon size progressively increases toward the easternmost sector, corresponding to the least populated areas. In contrast, smaller polygons are concentrated around densely populated urban centers, reflecting the largest densities of healthcare facilities in these areas. This spatial distribution not only reveals the impact of population density on the accessibility of healthcare services but also provides a clear and intuitive framework to understand how hospital services are distributed and their implications for different communities (Fig. 1C).

### Transportation network and road classification

The dataset, gathered from the *Spanish Center for Geographic Information* (CNIG)^50^, compiles the complete road infrastructure of the region. Roads are classified into five categories based on capacity and maximum allowed speed: highways and highways, multilane roads, conventional roads, urban roads, and rural roads. Estimated theoretical speeds align with legal limits: 120 km/h for roads, 100 km/h for multilane roads, 90 km/h for conventional roads, 50 km/h for urban roads, and 30 km/h for rural roads.

This dataset covers over 160,372 km of the road network, a figure significantly higher than the actual length due to the duplication of certain categories where one-way segments are represented separately. By category, highways account for 3.6% of the network, multilane roads 0.26%, conventional roads 20%, urban roads 11.5%, and rural roads 64.5%. The large proportion of rural roads reflects the widespread dispersion of the population. Data are provided in geospatial format, with 908,883 linear features representing the different road categories, maintaining similar proportions to their total length. Table 2 summarizes the dataset, detailing the number and total length of each category of roads, along with the number of polyline features for GIS analysis.

**Table 2:** Summary of the road infrastructure dataset

Hierarchy level	Road classes	Polyline features	Total length [km]	Max. speed [km/h]
1	High capacity roads	15,413	5813.60	120
2	Multilane road	3691	422.90	100
3	Regular road	226,469	32,100.16	90
4	Urban street	230,214	18,573.52	50
5	Rural road	433,096	103,461.95	30

### Assessing travel time accessibility

This subsection assesses the regional accessibility to hospitals based on travel times, using the current distribution as a baseline scenario (*Scenario S0*). We then examine the effects of progressively reducing the number of hospitals, from maintaining the full network to a critical reduction of up to 75%. In total, four scenarios are proposed (*Scenarios S1–S4*), through which we identify the optimal spatial configuration to enhance accessibility under each condition.

### Scenario S0

**Fig. 2:**
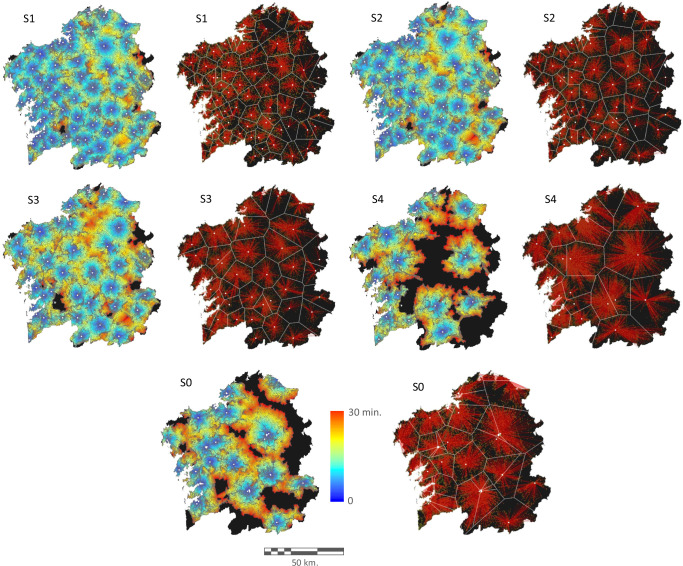
Travel-time accessibility to hospital facilities across all scenarios. *Scenario S0* represents the current state, while *Scenario S1* corresponds to an optimized configuration maintaining the same number of hospitals. While hospital facility numbers vary across scenarios, the number of population nodes remains constant. Although the networks visually represent Euclidean distances, actual distances are computed based on the road infrastructure. These panels also depict Voronoi boundaries, illustrating the number of hospitals and their theoretical service areas.

We assess the regional accessibility to the 79 officially documented hospitals, defined as *Scenario 0*. These hospitals are primarily concentrated in or near densely populated urban centers. Figure 2 shows current travel times based on the existing road network. Accessibility is represented through 1-minute isochrones up to a 30-min threshold, which serves as the *critical reference point*. A color gradient from blue (shorter travel times) to red (longer travel times) highlights variations within this range, while areas exceeding the 30-min threshold are shown in black.

The major cities and their metropolitan areas, along with several intermediate urban centers, demonstrate high accessibility. These intermediate cities include suburban hubs near large metropolitan areas and traditional capital cities serving surrounding rural populations. However, extensive regions, particularly in the east and south, as well as isolated pockets elsewhere, face limited accessibility, with travel times exceeding 30 min.

Almost half of the population lives within a 5-min drive to a hospital. However, accessibility varies by age: only 44.1% of elderly people fall within this range, compared to 48.8% of young people and 47.9% of adults. At the 30-min threshold, elderly people (4.3%) is disproportionately affected compared to young people (1.8%) and adults (2.6%). In absolute terms, 76,291 people, including 28,545 over 64 years-old, live beyond this critical limit. It is also worth noting that as travel times extend beyond 10 minutes, the proportion of the elderly population among the affected population increases.

### Scenario S1–S4

We assess regional accessibility by analyzing a series of potential scenarios. These scenarios, numbered *S1*–*S4*, involve progressively reducing the number of hospitals while optimizing their spatial configuration to minimize travel times. *Scenario S1* retains all 79 hospitals, but improves their distribution. *Scenarios S2*–*S4* gradually reduce capacity to 75%, 50%, and 25% of the current level, with 59, 40, and 20 hospitals, respectively. These changes impact both travel times and hospital service areas, as reflected in variations of the Voronoi diagram. Figure 2 shows a comparative analysis across scenarios.

Optimizing hospital locations in *Scenario S1* significantly improves accessibility by reducing critical areas and shrinking regions beyond the 30-minute threshold while expanding shorter travel times, as indicated by the increased presence of blue tones across the region. Only 0.1% of the total population (2314 individuals) and 0.2% of the elderly population (1082 individuals) remain beyond this threshold, with the largest improvements observed in intermediate areas outside major cities. However, low-accessibility areas predominantly persist in the central and eastern parts of the region.

As hospital numbers decline in subsequent scenarios, accessibility vulnerabilities become more pronounced in these areas. The provinces of Lugo and Ourense experience the most significant impacts, although other regions also see reductions in accessibility. Critical areas expand exponentially, and Voronoi polygons grow larger, reflecting increased distances to the nearest hospital.

An analysis by age composition highlights improvements in social equity. In the current configuration, the elderly population is disproportionately affected by longer travel times. *Scenario S1* reverses this trend for travel times greater than 20–25 min. Although subsequent scenarios introduce some fluctuations, the proportion of older adults in critical areas remains relatively stable at around 30–40%. This slightly exceeds their overall regional proportion, 28.7% in this dataset^44^—26.1% according to the official population census published by the *Galician Statistical Office*^46^—reinforcing a consistent pattern: As the distance from the hospital increases, the share of older residents also increases.

### Comparative accessibility across scenarios

**Fig. 3:**
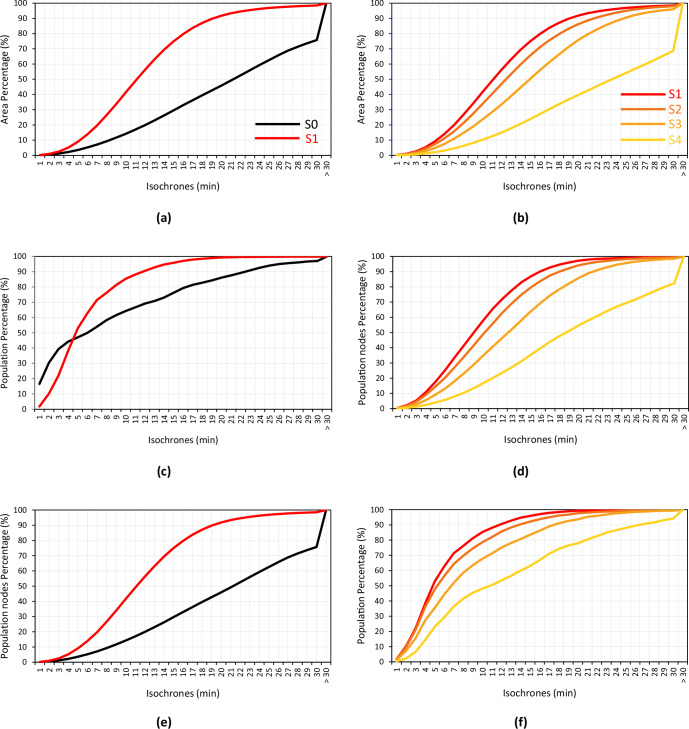
Comparative analysis of cumulative area, population nodes, and total population across scenarios. The *X*-axis represents travel times (in minutes), and the *Y*-axis displays the cumulative percentage of each indicator (area, population nodes, and total population) for each travel time interval. *Left panels:* Results for *Scenarios S0 and S1*. *Right panels:* Results for *Scenarios S1–S4*.

The optimization of hospital locations leads to substantial improvements in accessibility in all key indicators. Figure 3 presents aggregated data in 1-min travel time intervals, providing granular information on accessibility patterns. The results indicate that *Scenario S1* significantly improves accessibility compared to the current configuration (*Scenario S0*) by reducing the extent of critical-time areas and shortening travel times, allowing a larger fraction of the population to benefit from intermediate travel times.

The comparison between scenarios *S0* and *S1* highlights significant accessibility improvements in multiple dimensions. The total covered area expands by 22.8% (from 75.7% to 98.5%), the number of population nodes served increases by 10.9% (from 88.7% to 99.6%), and the total population reached increases by 2.8% (from 97.1% to 99.9%). These improvements are particularly pronounced at intermediate travel times. For example, at the 15-min mark, the covered area expands by 45.4% (from 29.6% to 75%), the number of population nodes increases by 40.9% (from 46.1% to 87%), and the total population served increases by 19.7% (from 76.2% to 95.9%). The only exception occurs within the first four minutes, where *Scenario S0* slightly outperforms *S1* in terms of total population coverage. However, beyond this threshold, *Scenario S1* consistently offers greater coverage in all indicators.

As the number of hospitals is progressively reduced from *Scenarios S1*–*S4*, accessibility declines, though the effects remain moderate in the early stages. *Scenarios* *S1*–*S3* maintain relatively stable performance with manageable reductions in service levels. However, in *Scenario S4*—where hospital numbers drop to just 25% of the current level—accessibility sharply deteriorates. The patterns in *S4* closely resemble those in *S0*, particularly in terms of area coverage and the number of population nodes served. This suggests that drastic reductions in hospital infrastructure lead to severe service degradation, reinforcing the importance of optimized spatial redistribution to ensure equitable access to healthcare.

**Fig. 4:**
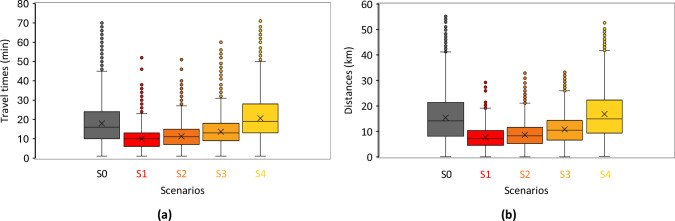
Box-and-whisker diagrams for all population nodes by province. The left column shows travel times (**a**), and the right column shows Euclidean distances (**b**). Results by scenarios are represented by rows, while results by provinces are displayed sequentially in ascending order within each individual chart.

A direct comparison of distances and travel times provides a more comprehensive analysis. Euclidean distances between each population node and the nearest hospital are estimated based on the spatial network topology defined for each scenario, as illustrated in Fig. 2. A summary of results for the entire region across all scenarios is presented in Fig. 4. *Scenarios* *S0* and *S4* exhibit strikingly similar performance, particularly in terms of distance, and both perform significantly worse than the intermediate scenarios. These findings highlight the crucial role of optimizing hospital distribution to improve healthcare access and minimize disparities.

### Regional and demographic disparities in accessibility

A provincial breakdown highlights substantial disparities in healthcare access, with Lugo and Ourense emerging as the most vulnerable regions. Under the current hospital distribution (*Scenario S0*), these provinces have the highest proportions of residents who live beyond the 30-min isochrone: 4.9% in Lugo and 6.4% in Ourense, compared to 3.5% in A Coruña and Pontevedra. The situation is even more critical for the elderly population, with 6.5% in Lugo and 9.5% in Ourense residing beyond this threshold. These figures far exceed those of other provinces, highlighting the increased accessibility challenges these regions face.

**Fig. 5:**
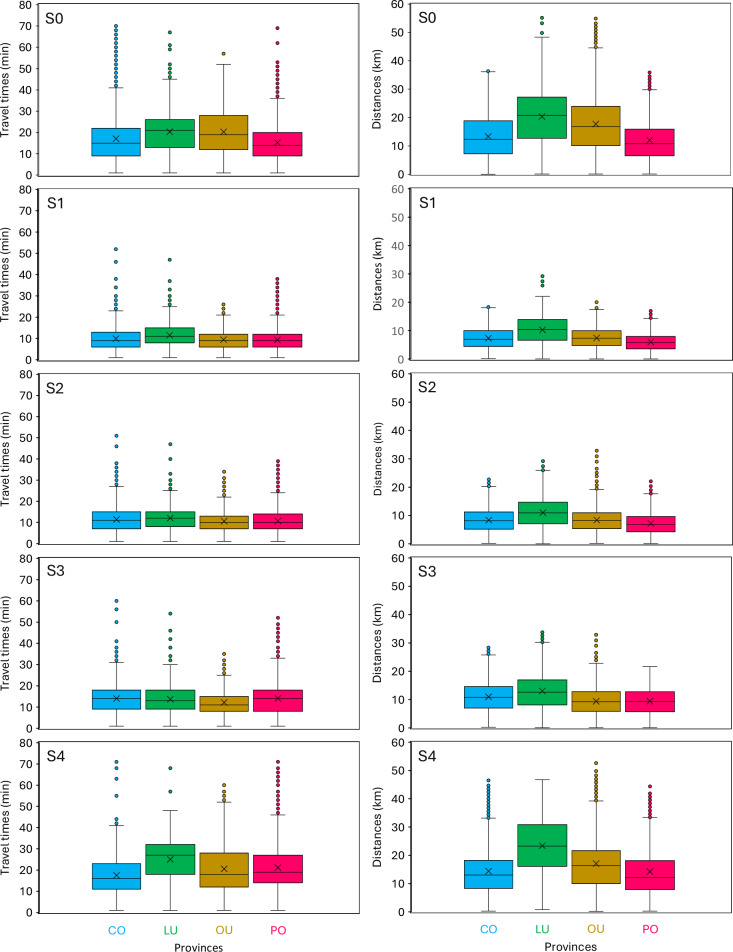
Box-and-whisker diagram for all population nodes by provinces. *Left:* Travel times; *Right:* Euclidean distances. Results for each scenario are displayed sequentially in ascending order.

Optimizing hospital locations in *Scenario S1* leads to a significant reduction in disparities in all provinces, with the most notable improvements observed in Lugo and Ourense. The fraction of the population living beyond the 30-minute threshold drops to ~0.1% in these provinces, while in A Coruña and Pontevedra, the residual values remain slightly higher, ranging from 0.2% to 0.4%. This trend is also observed in *Scenario S1*, which improves accessibility for older populations. Figure 5 shows that scenarios *S1* and *S2* consistently improve both distances and travel times in all provinces, promoting greater equity. *Scenario S3* continues this trend, with particularly noticeable improvements in Ourense. However, *Scenario S4* highlights vulnerabilities in all provinces, although A Coruña remains the least affected. Despite Lugo exhibiting the highest Euclidean distances in all scenarios, these distances do not always correlate with longer travel times, suggesting differences in network efficiency.

**Fig. 6:**
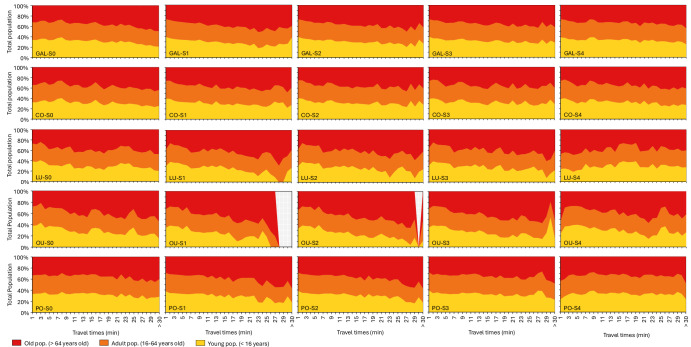
Distribution of the total population by age group across scenarios for the entire region and individual provinces. The *X*-axis represents travel times (minutes), while the *Y*-axis indicates the population percentage within each age group for each travel time interval. Results by administrative regions (i.e., Galicia or individual provinces) are represented by rows, while results by scenarios are displayed sequentially in ascending order across columns.

In all scenarios, elderly populations remain disproportionately represented in critical access areas. In Lugo province, 29.9% of the total population is elderly, compared to 31.9% in Ourense^46^, with slightly lower figures in the point grid dataset (28.2% and 31.1%, respectively)^44^. However, in *Scenario S0*, the proportion of elderly residents in critical areas increases to 37.6% in Lugo and 46.4% in Ourense, indicating the heightened vulnerability for this age group. Although no scenario fully eliminates this disparity, all optimized configurations result in significant reductions. Even in the most constrained scenario (*S4*), the proportion of elderly residents in critical areas drops to 33.1% in Lugo and 37.2% in Ourense. This demonstrates that an optimized redistribution of hospitals can significantly improve healthcare access for the elderly population, achieved solely through a reconfiguration of existing resources. Figure 6 offers a detailed breakdown of age-group disparities across all provinces and scenarios, while Table 3 provides a comprehensive summary of the supporting data and visualizations.

**Table 3:** Summary of results by travel times and distances for the entire region and each province

Region	Scenario	Hospitals	Distance (km)				Travel times (h)			
			Total	Mean per hosp.	Mean per pop. node	Mean per 10 people	Total	Mean per hosp.	Mean per pop. node	Mean per 1000 people
Galicia	S0	79	163,248.64	2066.44	15.48	0.62	3119.69	39.49	0.30	1.19
	S1	79	81,164.71	1027.40	7.70	0.31	1767.18	22.37	0.17	0.68
	S2	59	91,647.79	1553.35	8.69	0.35	1975.68	33.49	0.19	0.76
	S3	40	114,073.52	2851.84	10.82	0.44	2395.73	59.89	0.23	0.92
	S4	20	177,229.86	8861.49	16.81	0.68	3611.37	180.57	0.34	1.38
A Coruña	S0	29	52,355.74	1805.37	13.45	0.48	1097.79	37.85	0.28	1.00
	S1	27	28,612.00	1059.70	7.35	0.26	643.18	23.82	0.17	0.59
	S2	19	32,687.03	1720.37	8.89	0.30	740.08	38.95	0.19	0.67
	S3	11	42,830.66	3893.70	11.00	0.39	907.33	82.48	0.23	0.83
	S4	8	55,940.03	6992.50	14.37	0.51	1136.07	142.01	0.29	1.03
Lugo	S0	9	47,642.44	5293.60	20.36	1.64	793.25	88.14	0.34	2.73
	S1	14	24,103.14	1721.65	10.30	0.83	449.20	32.09	0.19	1.55
	S2	12	25,738.20	2149.02	11.02	0.89	471.30	39.28	0.20	1.62
	S3	9	30,546.57	3394.06	13.05	1.05	534.25	59.36	0.23	1.84
	S4	2	54,773.33	27,386.66	23.41	1.89	975.33	487.67	0.42	3.36
Ourense	S0	11	32,004.28	2909.48	17.72	1.10	610.82	55.53	0.34	2.10
	S1	17	13,375.95	786.82	7.41	0.46	285.12	16.77	0.16	0.98
	S2	12	15,153.16	1262.76	8.39	0.52	318.42	26.53	0.18	1.10
	S3	10	17,002.98	1700.30	9.41	0.58	365.63	36.56	0.20	1.26
	S4	4	30,809.29	7702.32	17.06	1.06	618.83	154.71	0.34	2.13
Pontevedra	S0	30	31,235.75	1041.19	12.48	0.33	617.55	20.59	0.25	2.12
	S1	21	15,063.49	717.31	6.02	0.16	389.47	18.55	0.16	1.34
	S2	16	18,009.21	1125.58	7.20	0.19	445.67	27.85	0.18	1.53
	S3	10	23,679.64	2367.96	9.46	0.25	588.18	58.82	0.23	2.02
	S4	6	35,691.31	5948.55	14.26	0.38	880.75	146.79	0.35	3.03

## Discussion

This study provides crucial information on the physical accessibility of healthcare facilities, especially highlighting regional disparities and their implications for healthcare equity.

Access to healthcare is unevenly distributed across regions, with persistent and significant spatial disparities consistently emerging. Hospitals are mainly concentrated in urban areas, ensuring better healthcare access for city residents while placing rural and remote populations at a disadvantage. In particular, 76,292 individuals, representing 2.9% of the total population, live beyond the 30-min travel threshold identified as critical in this study. This threshold evidences the structural challenges involved in achieving equitable healthcare access, particularly for populations in remote and underserved regions where spatial and infrastructural limitations persist.

These spatial disparities are closely related to sociodemographic factors, with vulnerable populations, especially the elderly, being disproportionately affected. In this study, we found that 28,546 people over the age of 64 live beyond the 30-min threshold, representing 4.3% of the total elderly population residing in these critical areas. This group represents 37.4% of all the people residing in critical areas, significantly larger than their 26.1% share of the total population. This underscores the increased vulnerability of the elderly population regarding physical access to healthcare services.

Optimizing hospital distribution can dramatically improve accessibility for both the general population and specific age groups. In the optimized model, the proportion of the total population in critical areas drops to just 0.1%, and the share of elderly residents in these areas is reduced to 0.2%. However, the effectiveness of optimization varies regionally. Intermediate optimization scenarios yield better outcomes in Ourense, where the population is more concentrated, with a total of 1806 population nodes, compared to Lugo, which exhibits a more dispersed settlement pattern with 2340 population nodes. These differences in results stem from the contrasting spatial distribution of the population across provinces. Therefore, optimization strategies should be context-specific and tailored to regional demographic and spatial characteristics in order to maximize their effectiveness.

Simulating scenarios with reduced hospital facilities further emphasizes the vulnerabilities of certain regions. Although optimized models improve hospital distribution, a reduction in the number of hospitals disproportionately affects specific areas, revealing structural weaknesses in the healthcare network. This underscores the need for careful evaluation of hospital facility reductions in healthcare infrastructure planning.

The increase in the number of hospitals does not necessarily lead to improved regional accessibility. The study demonstrates that the current hospital distribution performs much worse than in a scenario where the number of hospitals is reduced by 50% achieving results similar to those seen with a reduction of 75% in hospital facilities. This counterintuitive finding suggests that hospital distribution strategies must balance both quantity and optimal location to be effective.

Effective hospital redistribution should take into account both quantitative factors, such as population size, and qualitative factors, such as age composition and dependency levels. Prioritizing these factors is particularly important to ensure that elderly and vulnerable populations in remote areas have access to the necessary healthcare services.

Optimized spatial configurations should match hospital capacity to the structure and properties of the spatial network, which are determined by road infrastructure. The study reveals significant heterogeneity in the number of links and the population associated with each potential hospital location, highlighting the need for context-specific adjustments.

Healthcare managers, spatial planners, and policymakers face the critical challenge of optimizing hospital locations to balance resource efficiency with equitable accessibility, particularly in terms of minimizing travel times for the population. Although placing hospitals in urban centers benefits most residents, it often reduces access for those in underserved areas, particularly elderly populations, who require frequent and specialized care. These trade-offs can be analyzed through network science, which offers insight into the costs and benefits of different hospital distribution models. Increasing the number of hospitals generally reduces travel times, but also raises infrastructure and operational costs. In contrast, reducing the number of hospitals may seem cost-effective, but often results in longer travel distances and higher transportation expenses^51^. In this study, comparing optimized networks with 79 and 20 hospitals (scenarios *S1* and *S4*) demonstrates that reducing facilities more than doubles both travel times (*Scenario S1*: 1767 hours; *Scenario S4*: 3611 hours) and total distances traveled (*Scenario S1*: 81,165 km; *Scenario S4*: 177,230 km). Therefore, to maintain care levels despite a reduction in the number of hospitals, health policymakers often rely on costly transport solutions, such as emergency aerial transfers, which have become increasingly frequent in this region^52^. These measures inevitably involve significant long-term costs.

An optimal hospital distribution prioritizes social justice and spatial equity, advocating for strategic relocations of hospitals to underserved regions. Placing healthcare facilities outside dense urban centers not only improves accessibility but also promotes local development, alleviates urban agglomeration pressures, and encourages decentralized urbanization. This approach improves the alignment of services with population needs and reduces transportation costs.

Geographic factors play an essential role in shaping settlement patterns and accessibility disparities. Misalignments between Voronoi diagrams and the actual spatial network arise from topography and road layout, which highlight discrepancies between Euclidean distances and actual travel times. Urban-adjacent areas, for example, may not always be easily accessible due to these factors, as seen in the westernmost sector of the region^53,54^.

Social dynamics and evolving behaviors often develop as adaptive responses to the barriers in accessibility. Many elderly people relocate closer to hospitals, improving their accessibility and helping reduce vulnerability in critical areas. However, sparsely populated regions can attract younger populations, particularly with the rise of teleworking, as people seek alternatives to living in urban centers. These demographic changes alter the age distribution and reshape the spatial demand for healthcare services. Addressing these complexities requires adaptive hospital distribution strategies that can accommodate evolving spatial trends. This challenges traditional spatial planning paradigms and introduces the concept of *transient hospitals*—facilities capable of adjusting their locations to meet short- or medium-term demand—. This flexibility aligns with the dynamic nature of globalization and emphasizes the need for interdisciplinary collaboration between healthcare managers, policymakers, spatial planners, and transportation engineers.

Although this study provides valuable insights, it is important to recognize several limitations that may influence the findings. One key assumption is that urban hospitals complement rather than compete with each other, given that many specialize in specific medical fields. This may oversimplify the complexity of hospital networks, where overlapping services can occur. The analysis also makes some simplifications that could affect the results, such as assuming uniform connectivity across the road network, which may overlook variations in road conditions, and considering the region as a closed system, disregarding interactions with neighboring areas. Additionally, potential hospital sites are positioned on a regular grid, which may not fully reflect real-world spatial constraints. Although the spatial interpolation methods used are robust, some parameters—such as the number of nodes, distance thresholds, and decay functions—were simplified, potentially impacting the outcomes. Despite these limitations, the assumptions provide a solid foundation for the analysis.

Building upon these insights, this study underscores the critical importance of ensuring equitable access to healthcare for all individuals, regardless of their geographic location, as a key factor in promoting social justice and territorial convergence. Although the analysis focuses on a specific region with unique sociodemographic characteristics, its findings are of broader relevance for areas facing aging populations and uneven demographic distributions^55^. Despite certain simplifications—including the assumption of universal access to healthcare in Spain and the exclusion of socioeconomic disparities among individuals—the results emphasize the urgent need for sustainable healthcare access policies that reduce disparities and ensure efficient and inclusive service provision across diverse spatial contexts. Future research should expand the study area, include real-world traffic conditions, and improve hospital placement to better match capacity with demand and strengthen healthcare access and policy impact.

## Methods

The methodology is structured into two stages. In the first stage, we use a GIS-based approach to estimate the travel times from each population settlement to the nearest hospital in the region. This step assesses current accessibility in terms of travel times, establishing what is referred to as *Scenario S0*. In the second stage, a spatial reallocation model is developed to optimize the location of hospital facilities, improving travel time accessibility to hospitals. This process explores multiple spatial configurations based on the number of facilities, leading to a series of hypothetical scenarios (*S1*–*S4*). All simulations and calculations are performed using ArcGIS software (version 10.8), developed by the *Environmental Systems Research Institute* (ESRI).

Of the 10,545 available population nodes, 6356 contain age composition data for the three defined age groups. The remaining 4189 nodes have missing data for at least one age group, specifically for the young population (absent in all these nodes), 995 lack data for adults and 3364 lack data for the elderly. To address these gaps, we implement a spatial interpolation process in three steps, each targeting an age group. The number of input nodes varies: 6356 for the young, 9550 for adults, and 7181 for the elderly.

We evaluate two interpolation methods: *Inverse distance weighting* (IDW) and *Ordinary Kriging* based on their suitability and robustness. *Inverse distance weighting* (IDW) is a deterministic method that averages nearby values, with distances weighted by a quadratic decay function to prioritize closer nodes^56,57^. *Ordinary Kriging* uses a variogram to model spatial autocorrelation and estimate uncertainty, offering confidence intervals^58,59^. Both methods are applied with two spatial constraints: a maximum influence of 12 neighboring nodes and a distance threshold of 10 km. We observed that both methods provide similar accuracies for estimating the missing data; therefore, we opted to use the IDW method.

Using the IDW method, we derive the age composition values for each node based on the data from neighboring nodes. For a point *z* with an unknown value at a specific location *x*, the estimated value can be calculated as a weighted average of observed values from nearby nodes. The weights are inversely proportional to the distance between the location *x* and the observed points *x*_*i*_. Mathematically, the estimated value $\hat{z}(x)$ is expressed as

3 $${\hat{z}}(x)=\frac{\mathop{\sum}\nolimits_{i=1}^{n}{w}_{i}{z}_{i}}{\mathop{\sum}\nolimits_{i=1}^{n}{w}_{i}}$$

where *w*_*i*_ represents the weight assigned to each observation *z*_*i*_, calculated as

4 $${w}_{i}=\frac{1}{d{(x,{x}_{i})}^{\beta }}$$

where *d*(*x*, *x*_*i*_) represents the Euclidean distance between the location *x* and the observation *x*_*i*_, and *β* is a positive parameter that determines the rate at which the weight decreases with distance. This approach prioritizes the closer points, assigning them greater influence in the estimation process. We tested different values of *β* within the range between 1 and 3 and decided to set *β* = 2 as the most widely accepted in the literature^60^.

The interpolation process generates percentage estimates for each age group, which are then multiplied by the known total population per node to obtain absolute values. To assess the accuracy of the estimates, we conduct a cross-validation using 4189 nodes with partially available official data. The results show a 96% adjustment rate, validating the precision and consistency of the interpolation methods in different scenarios. This approach successfully fills data gaps, completes the data set for all 10,545 population nodes, and ensures comprehensive spatial coverage.

### Scenario S0

We construct a spatial network linking demand (population nodes) and supply (hospitals) by geolocating each node. The population nodes correspond to the centroids of grid cells, typically spaced at 1 km^2^, though smaller in peripheral areas due to grid adjustments. The network comprises 10,545 population nodes and 79 georeferenced hospitals, connected via existing road infrastructure under ideal travel conditions based on road capacity, hierarchy, and characteristics. Although this assumption generally holds for emergency vehicles, certain segments (e.g., urban roads during peak hours) may introduce deviations.

Within a GIS framework, population nodes and hospital facilities are represented as point features, while roads are represented as linear features. Travel times are computed by evaluating all possible paths (360°) and selecting the shortest. Key assumptions include: (a) topography influences road design but is not explicitly modeled, (b) a fuzzy tolerance of 1 km ensures connectivity, and (c) the network is fully connected. Accessibility is visualized using an isochrone map, where each isochrone delineates areas that can be reached in a given travel time, shaped by the structure of the road network. Following previous studies^9,33,61^, we adopt a critical response time of 30 min. Areas below this threshold are color-graded from blue to red (indicating decreasing accessibility), while those exceeding 30 min appear in black. Finally, we quantify the total and age-specific populations within each hospital accessibility band.

### Scenario S1–S4

We propose a spatial redistribution of healthcare facilities to optimize the provision of services in alternative scenarios. *Scenario S1* offers a reconfiguration of existing hospitals (*Scenario S0*) without changing their total number, to maximize health coverage. *Scenarios S2–S4* gradually reduce the number of hospitals, from 25% (*Scenario S2*) to 75% (*Scenario S4*) to assess the resilience of the network in conditions of potential crises and demographic shifts, including aging, migration, and depopulation^62–64^.

To estimate the optimal distributions of the facilities, we employ the *Minimize Impedance* algorithm, an adaptation of *Hillsman’s p-median* approach that integrates a substitution heuristic of the vertex^65^ for iterative optimization^66,67^. The algorithm seeks to minimize *total impedance*, defined as

5 $$I=\mathop{\sum }\limits_{i=1}^{n}{c}_{i}$$

where *c*_*i*_ represents the impedance (for example, travel time) associated with each network segment. In facility allocation, the objective is to minimize the cumulative impedance between demand points *x*_*i*_ and supply centers *y*_*j*_:

6 $$I=\mathop{\sum }\limits_{i=1}^{m}\mathop{\sum }\limits_{j=1}^{n}{w}_{ij}{c}_{ij}$$

where *c*_*ij*_ is the impedance between the demand point *x*_*i*_ and the supply center *y*_*j*_, and *w*_*ij*_ is a weight representing the relative importance of *x*_*i*_ to *y*_*j*_.

To estimate the optimal locations for regional hospitals, we build a dense grid of 5 × 5 km cells, constrained by administrative boundaries, to ensure spatial precision and complete coverage. Cells smaller than 15 km^2^ are excluded, resulting in 1163 candidate hospital locations. The impedance is based on travel times and incorporates the characteristics of the road and the capacity. A weighting criterion accounts for both population size and aging, treating aging as a vulnerability factor.

7 $${w}_{i}={{T{P}}}_{i}\cdot {{A{I}}}_{i}$$

where *TP*_*i*_ is the total population at node *i*, and *AI*_*i*_ is the aging index:

8 $${{A{I}}}_{i}=\frac{{{E{P}}}_{i}}{{{Y{P}}}_{i}}$$

with *EP*_*i*_ representing the elderly population (65+ years) and YP_*i*_ representing the young population (under 16 years).

The algorithm identifies optimal hospital placements by minimizing travel times while maximizing coverage using the weighting factor in the equation. Each scenario yields a unique allocation that balances accessibility and efficiency.

## Data Availability

The primary datasets used in this study are publicly available in the repositories cited in the references. The datasets generated and/or analysed during the current study are available from the corresponding author upon reasonable request.
